# HCV-Associated Exosomes Upregulate RUNXOR and RUNX1 Expressions to Promote MDSC Expansion and Suppressive Functions through STAT3–miR124 Axis

**DOI:** 10.3390/cells9122715

**Published:** 2020-12-18

**Authors:** Bal Krishna Chand Thakuri, Jinyu Zhang, Juan Zhao, Lam N. Nguyen, Lam N. T. Nguyen, Madison Schank, Sushant Khanal, Xindi Dang, Dechao Cao, Zeyuan Lu, Xiao Y. Wu, Yong Jiang, Mohamed El Gazzar, Shunbin Ning, Ling Wang, Jonathan P. Moorman, Zhi Q. Yao

**Affiliations:** 1 Center of Excellence for Inflammation, Infectious Disease and Immunity, James H. Quillen College of Medicine, East Tennessee State University, Johnson City, TN 37614, USA; thakuri@etsu.edu (B.K.C.T.); zhangj2@etsu.edu (J.Z.); zhaoj2@etsu.edu (J.Z.); nguyenl@etsu.edu (L.N.N.); nguyenln@etsu.edu (L.N.T.N.); niecem@etsu.edu (M.S.); khanals@etsu.edu (S.K.); dangx1@etsu.edu (X.D.); caod01@etsu.edu (D.C.); LUZ001@mail.etsu.edu (Z.L.); WUXY@etsu.edu (X.Y.W.); JIANGY2@mail.etsu.edu (Y.J.); ELGAZZAR@etsu.edu (M.E.G.); nings1@etsu.edu (S.N.); wangl3@etsu.edu (L.W.); moorman@etsu.edu (J.P.M.); 2 Division of Infectious, Inflammatory and Immunologic Diseases, Department of Internal Medicine, Quillen College of Medicine, ETSU, Johnson City, TN 37614, USA; 3 Hepatitis (HCV/HBV/HIV) Program, James H. Quillen VA Medical Center, Department of Veterans Affairs, Johnson City, TN 37614, USA

**Keywords:** HCV, immune suppression, MDSCs, miR124, RUNXOR, RUNX1

## Abstract

RUNX1 overlapping RNA (RUNXOR) is a long non-coding RNA and plays a pivotal role in the differentiation of myeloid cells via targeting runt-related transcription factor 1 (RUNX1). We and others have previously reported that myeloid-derived suppressor cells (MDSCs) expand and inhibit host immune responses during chronic viral infections; however, the mechanisms responsible for MDSC differentiation and suppressive functions, in particular the role of RUNXOR–RUNX1, remain unclear. Here, we demonstrated that RUNXOR and RUNX1 expressions are significantly upregulated and associated with elevated levels of immunosuppressive molecules, such as arginase 1 (Arg1), inducible nitric oxide synthase (iNOS), signal transducer and activator of transcription 3 (STAT3), and reactive oxygen species (ROS) in MDSCs during chronic hepatitis C virus (HCV) infection. Mechanistically, we discovered that HCV-associated exosomes (HCV-Exo) can induce the expressions of RUNXOR and RUNX1, which in turn regulates miR-124 expression via STAT3 signaling, thereby promoting MDSC differentiation and suppressive functions. Importantly, overexpression of RUNXOR in healthy CD33^+^ myeloid cells promoted differentiation and suppressive functions of MDSCs. Conversely, silencing RUNXOR or RUNX1 expression in HCV-derived CD33^+^ myeloid cells significantly inhibited their differentiation and expressions of suppressive molecules and improved the function of co-cultured autologous CD4 T cells. Taken together, these results indicate that the RUNXOR–RUNX1–STAT3–miR124 axis enhances the differentiation and suppressive functions of MDSCs and could be a potential target for immunomodulation in conjunction with antiviral therapy during chronic HCV infection.

## 1. Introduction

Hepatitis C virus (HCV) can employ different strategies to evade host immunity and harness virus persistence, thus serving as an excellent model for studying the mechanisms of virus-mediated host immune dysfunction and viral persistence in humans [1,2]. While direct-acting antivirals (DAA) can efficiently clear HCV infection in the majority of treated individuals, this therapeutic cocktail faces new issues, such as viral mutation, relapse, and reinfection after treatment [3,4]. In addition, “virological cure” does not always lead to “immunological cure”, and some immune disorders persist after DAA treatment with sustained virological response (SVR) [5]. The failure to manage many chronic infectious diseases, including HCV, stems from our incomplete understanding of the pathogen–host interactions that can dysregulate host immune responses.

Myeloid-derived suppressor cells (MDSCs) are a heterogeneous population of immature myeloid cells produced during aberrant myelopoiesis under pathogenic conditions, such as cancer and inflammatory or infectious diseases [6,7,8]. MDSCs have gained special attention due to their role in suppressing host immune responses [9,10]. MDSCs contribute to immune homeostasis via limiting excessive inflammatory processes, but their expansion may be at the expense of pathogen elimination, resulting in persistent infection [8]. We and others reported that MDSCs expand and inhibit T cell functions in multiple disease models, including chronic viral (HCV and HIV) infections [11,12,13,14,15,16,17,18]. However, the mechanisms responsible for MDSC differentiation and suppressive functions during viral infection remain unclear.

Long non-coding RNAs (lncRNAs) are genomic transcripts of non-coding RNAs consisting of both lncRNA (>200 nt in length) and miRNAs (~20 nt) and possess regulatory functions [19,20,21]. Thus far, thousands of lncRNAs have been discovered, but most of their functions have not been characterized [22]. Recent studies suggest that lncRNAs are involved in almost all cellular processes via a variety of mechanisms [23,24,25,26,27], and their expressions are species-, cell-, and disease-specific [19,20,21].

RUNXOR is a 260-kb un-spliced lncRNA that interacts epigenetically with multiple sites within the RUNX1 locus [28]. RUNX1 is a tumor suppressor gene responsible for modulating various hematopoietic regulators. RUNXOR regulates the expression of RUNX1 in acute myeloid leukemia (AML) cells by binding to its promoter and enhancer [29]. It may also be involved in chromosomal translocation that normally occurs during malignancies [28]. RUNXOR can directly bind to chromatin, resulting in the formation of a long-range intra-chromosomal loop, which is a typical epigenetic mechanism employed by a regulatory element so that it can regulate expression of genes that are distantly located [29,30]. Recently, the RUNXOR–RUNX1 axis has been shown to be associated with the development of MDSCs in lung cancer [30].

Exosomes are membrane-bound extracellular microvesicles that serve as carriers to transfer various signaling molecules (such as viral RNA, mRNA, or ncRNA) between cells without any direct cell-to-cell contact, and play an important role in regulating immune responses [31,32,33,34]. Exosomes are produced and secreted by all types of cells and importantly, exhibit enrichment of the human tetraspanin CD81, a receptor for the HCV E2 glycoprotein [35]. Infected hepatocytes release HCV genomic materials into the peripheral blood in the form of circulating exosomes that exploit the fusogenic capabilities of exosomes with other cells, transmitting HCV-RNA and thereby dysregulating host immune responses, even in the presence of neutralizing antibodies [36,37,38]. We previously showed that exosomes isolated from the plasma of HCV patients contain HCV-RNAs and were able to promote MDSC expansion thereby inhibiting T cell function [18]. Whether HCV RNA-associated exosomes (HCV-Exo) can promote MDSC expansion and suppression through regulation of RUNXOR–RUNX1 expression has yet to be determined.

In this study, we characterized the expression and role of RUNXOR and RUNX1 in MDSC expansion and function during HCV infection. We demonstrated that the expressions of RUNXOR and RUNX1 are upregulated and play a role in driving MDSC expansion through regulating STAT3 and miR-124 expressions during HCV infection. We also discovered that HCV-Exo dysregulate the RUNXOR–RUNX1–STAT3–miR-124 axis, thus playing an important role in regulating MDSC expansion and their immunosuppressive functions. Our study reveals a novel mechanism of immune dysregulation during chronic viral infection.

## 2. Materials and Methods

### 2.1. Subjects

The study subjects were composed of two populations: 50 chronically HCV-infected individuals and 54 healthy subjects (HS). HCV genotype (70% type 1, 30% type 2 or 3) and viral load (ranging 17,000–17,000,000 IU/mL) were determined by Lexington VAMC, and all subjects were virologically and serologically positive for HCV prior to antiviral treatment. Healthy subjects were negative for HBV, HCV, and HIV infections and were recruited by BioIVT (Gray, TN, USA). The characteristics of the subjects recruited in this study are described in Table 1.

### 2.2. Cell Isolation, Culture, and Flow Cytometric Analysis

Peripheral blood mononuclear cells (PBMCs) were isolated from whole blood using Ficoll density gradients (GE Healthcare, Piscataway, NJ, USA). CD33^+^ cells were isolated from PBMCs using a CD33^+^ Cell Isolation Kit and a MidiMACS™ Separator column (Miltenyi Biotec, Auburn, CA, USA). The cells were cultured in RPMI 1640 medium with 10% fetal bovine serum (Atlanta Biologicals, Flowery Branch, GA, USA), 100 IU/mL penicillin, and 2 mM L-glutamine (Thermo Scientific, Logan, UT, USA) at 37 °C and 5% CO_2_ atmosphere. Cell depletion and culture with exosomes were carried out as described previously [15,16,17,18]. Flow cytometry analysis of cell phenotypes and intracellular cytokines in PBMCs was carried out as described previously [16,17,18]. Anti-CD4-FITC, anti-IFN-γ-PE, anti-CD33-PE, anti-CD14-APC (Biolegend), anti-HLA-DR-FITC, anti-CD3-APC, anti-Arg1-PE, anti-pSTAT3-PerCP (all from Biolegend, San Diego, CA, USA) and anti-iNOS-PE (Novus biologicals, Centennial, CO, USA) reagents were used along with isotype control antibodies (BD Bioscience, San Jose, CA, USA). Levels of reactive oxygen species (ROS) in myeloid cells were measured using the H2DCFDA-based kit (Invitrogen, Carlsbad, CA, USA) according to the manufacturer’s protocol. The stained cells were acquired on an AccuriTM C6 flow cytometer (BD, Franklin Lakes, NJ, USA) and analyzed using FlowJo software (Tree Star, Ashland, OR). Isotype control antibodies (eBioscience, San Diago, CA, USA) and fluorescence minus one (FMO) controls were used to determine the background levels of staining and adjust multicolor compensation as a gating strategy.

### 2.3. Exosome Isolation and Purification

Plasma was purified from 50 mL of whole blood from the research subjects and filtered to exclude particles larger than 0.8 μm, using syringe filters (Millex-AA Cat #: SLAA033SS, Millipore, Billerica, MA, USA). Exosomes were then isolated from plasma by a differential centrifugation method as previously described [18].

### 2.4. lncRNA and miRNA Arrays and RT-qPCR Validation

CD33^+^ myeloid cells were purified from PBMCs as described above. Total cellular RNA from CD33^+^ cells (pooled from 6 chronic HCV patients and 6 HS) was isolated using the miRNeasy Mini kit (Qiagen, Valencia, CA, USA). The RNA quality and quantity were analyzed using a BioPhotometer spectrophotometer UV/VIS, and RNA integrity was determined using gel electrophoresis. LncRNAs were analyzed using the Arraystar gene array service (Rockville, MD. The miScript miRNA array was performed by Qiagen [17]. To validate the results for up- or downregulated miRNAs by real-time RT-PCR, cDNA was generated from total RNA by the Taqman advanced miRNA cDNA synthesis kit and the High-Capacity cDNA Reverse Transcription Kit (Thermo Scientific, Logan, UT, USA). The miRNA expression levels were assessed by RT-qPCR using Taqman^®^ fast advanced master mix (Thermo Scientific) and the CFX96TM RT-PCR Detection System (Bio-Rad Laboratories, Hercules, CA, USA). The miRNA levels were determined using the 2^−ΔΔct^ relative quantification method and were normalized to U6 RNA (SNORD61) level as an internal control. 

### 2.5. Transfection and Co-Culture Experiments

For miR-124 inhibition, CD33^+^ cells isolated from PBMCs of HS were transfected with 30 pmol of miR-124 inhibitor or negative control inhibitor. For miR-124 overexpression, CD33^+^ cells isolated from PBMCs of HCV subjects were transfected with 30 pmol of miR-124 mimic or negative control mimic. For STAT3 knockdown, the CD33^+^ cells were transfected with 50 nM of a STAT3 SMART pool of siRNAs or a pool of scrambled siRNAs. For RUNXOR and RUNX1 knockdown, the cells were transfected with 50 nM of a RUNXOR SMART pool siRNAs, a RUNX1 SMART pool siRNAs, or a pool of scrambled siRNA (Lafayette, CO, USA). RUNXOR was overexpressed by using a Cas9-gRNA-RUNXOR promoter targeting vector and a RUNXOR Arm-pCMV-puromycin donor vector (kindly provided by Dr. Hu, Stanford University Medical School, CA). The transfection of miRNA or siRNA was performed using the Human Monocyte Nucleofector Kit and Nucleofector II Device (Lonza, Allendale, NJ, USA) following the manufacturer’s instructions. The transfected cells were cultured for two days in IMEM medium (Lonza) with 10% FBS. The cells were analyzed by flow cytometry or RT-qPCR as described above. For CD4^+^ T cell co-culture, autologous CD4^+^ T cells were stimulated with anti-CD3 (1 µg/mL) and anti-CD28 (2 μg/mL (BD Bioscience) for two days in IMEM complete medium, followed by adding the transfected CD33^+^ cells (at 1:2 ratio) for another three days.

### 2.6. Statistical Analysis

The parametric data are presented as mean ± SEM. Comparisons between two groups were analyzed using the unpaired *t*-test with Welch’s correction after checking the value of the F-test. One-tail paired *t*-test was used to compare two groups, and their associations were analyzed by Pearson correlation. The nonparametric data are presented as median with interquartile range and were analyzed by a one-tail Mann–Whitney test and then analyzed by Spearman correlation. *p*-values < 0.05 or *p* < 0.01 were considered significant or very significant, respectively.

## 3. Results

### 3.1. MDSCs Accumulate in Peripheral Blood during Chronic HCV Infection

**Figure 1:**
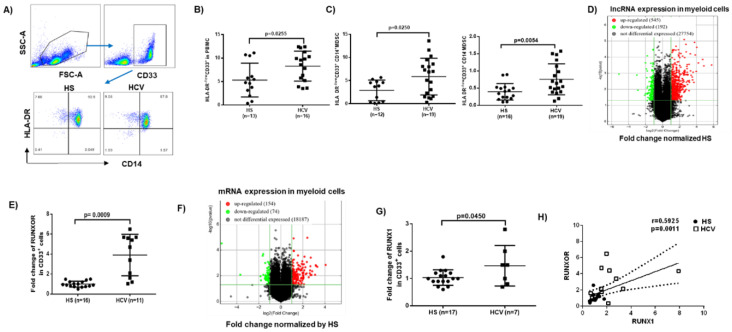
RUNXOR and RUNX1 are upregulated in MDSCs from HCV-infected individuals. (**A**) Representative dot plots of gating strategy for CD33^+^, and then HLA-DR^−^ and CD14^+/−^ cells in PBMCs. (**B**) Expansion of immature myeloid cells (HLA-DR^−/low^CD33^+^). (**C**) M-MDSCs (HLA-DR^−/low^CD33^+^CD14^+^ cells) and G-MDSCs (HLA-DR^−/low^CD33^+^CD14^−^ cells) in PBMCs from HCV-infected individuals compared with HS, as determined by flow cytometry. (**D**) Scatter plot of the heat map of lncRNA expression in CD33^+^ cells isolated from HCV-infected individuals versus HS (n = 6 per group). (**E**) RUNXOR expression in CD33^+^ cells isolated from HCV-infected individuals versus HS, as determined by real-time RT-qPCR. (**F**) Scatter plot of the heat map of mRNA expression in CD33^+^ cells isolated from HCV-infected individuals versus HS (n = 6 per group). (**G**) RUNX1 expression in CD33^+^ cells isolated from HCV-infected individuals versus HS, as determined by real-time RT-qPCR. (**H**) Pearson Correlation analysis of RUNXOR and RUNX1 expressions in CD33^+^ cells derived from the same subjects.

MDSCs play an important role in disease progression by suppressing host immune responses [6,7,8,39]. Human MDSCs are immature myeloid cell phenotyped as CD33^+^HLA-DR^−/low^, which can be further categorized into monocytic MDSCs (M-MDSCs) and granulocytic MDSCs (G-MDSCs) based on the level of expression of the monocytic marker CD14 [40,41]. To investigate the role and mechanisms of MDSC differentiation and functions during viral infection, we analyzed the frequencies of MDSCs within PBMCs from patients with chronic HCV infection compared to HS using flow cytometry. We found that the frequencies of total MDSCs (CD33^+^HLA-DR^−/low^), M-MDSCs (CD33^+^HLA-DR^−/low^ CD14^+^), and G-MDSCs (CD33^+^HLA-DR^−/low^ CD14^−^) were significantly increased in PBMCs in individuals with chronic HCV infection (Figure 1A–C).

### 3.2. RUNXOR and RUNX1 Are Upregulated in MDSCs during Chronic HCV Infection

To determine whether lncRNAs play a role in MDSC expansion during HCV infection, we analyzed the transcripts of lncRNAs and messenger RNAs (mRNAs) in MDSCs isolated from HCV-infected individuals and HS using the Arraystar gene expression array. Among the lncRNAs analyzed (shown as scatter plot in Figure 1D), 545 lncRNAs (red dots) were upregulated (>2-fold), 192 lncRNAs (green dots) were downregulated (>2-fold), and 27,754 lncRNAs (black dots) remained unchanged in MDSCs from HCV patients compared to HS. Given the critical role of RUNXOR in myeloid cell maturation [28,29,30], we further analyzed RUNXOR expression in the array and validated the results by real-time RT-qPCR, which revealed a >4-fold increase in MDSCs derived from HCV-infected individuals compared to HS (Figure 1E).

For mRNA expression analysis (shown as scatter plot in Figure 1F), 154 mRNAs (red dots) were upregulated, 74 mRNAs (green dots) were downregulated, and 18,187 mRNA transcripts remained unchanged. Notably, the mRNA array analysis revealed an upregulation of RUNX1 in myeloid cells derived from HCV subjects, which was validated by RT-qPCR assay (Figure 1G). Importantly, the expression levels of RUNXOR and RUNX1 positively correlated with each other, as determined by Spearman correlation (Figure 1H). Taken together, these results suggest that expressions of RUNXOR and its target gene RUNX1 are concurrently upregulated and may serve as a biomarker for MDSC expansion during chronic HCV infection.

### 3.3. Immunosuppressive Molecules Are Elevated in MDSCs during Chronic HCV Infection

**Figure 2:**
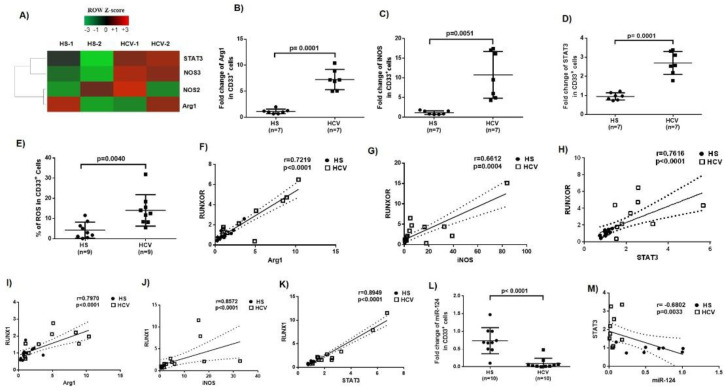
Upregulation of immunosuppressive molecules in MDSCs during HCV infection. (**A**) mRNA array showing upregulation of suppressive molecules (Arg1, NOS2, NOS3, and STAT3) in MDSCs from HCV-infected individuals. (**B**–**D**) Levels of Arg1, iNOS, and STAT3 gene expressions in CD33^+^ cells isolated from HCV-infected individuals versus HS, analyzed by real-time RT-qPCR. (**E**) ROS production in CD33^+^ cells derived from HCV-infected individuals versus HS, analyzed by the H2DCFDA assay. (**F**–**K**) The relationship between RUNXOR or RUNX1 and Arg1, iNOS, or STAT3 expression levels, analyzed by Pearson Correlation analysis. (**L**,**M**) Expressions of miR-124 in CD33^+^ cells from HCV-infected individuals versus HS, and correlation of miR-124 levels with STAT3 expression in these cells.

MDSCs suppress host immune responses by producing immunosuppressive mediators, such as arginase 1 (Arg1), inducible nitric oxide synthase (iNOS), signal transducer and activator of transcription 3 (STAT3), and reactive oxygen species (ROS) [17,18,19,20,42,43]. To determine the molecular mechanisms by which MDSCs exert their immunosuppressive effects during HCV infection, we measured the mRNA levels of these molecules that are implicated in myeloid cell differentiation and functions. Gene array analysis showed upregulation of STAT3, NOS3, NOS2, and Arg1 levels in MDSCs isolated from HCV patients versus HS (Figure 2A). These findings were validated by RT-qPCR, which revealed a 7-fold increase in Arg1 (Figure 2B), a 10-fold increase in iNOS (Figure 2C), and a 2.5-fold increase in STAT3 (Figure 2D). In addition, a significant increase in ROS production was detected using flow cytometry analysis using the H_2_DCFDA-based kit (Figure 2E). Notably, the elevated levels of Arg1, iNOS, and STAT3 showed positive correlations with both RUNXOR (Figure 2F–H) and RUNX1 expressions (Figure 2I–K), suggesting that HCV-induced MDSC expansion and upregulation of these immunosuppressive molecules may occur through the RUNXOR–RUNX1 axis during HCV infection.

### 3.4. MiR-124 Expression Negatively Correlates with STAT3 Levels in MDSCs during HCV Infection

We and others previously showed that miRNAs are involved in myelopoiesis orchestrated through the expressions of cytokine receptors and transcription factors [8,23,44,45]. To identify specific miRNAs that could affect myelopoiesis during HCV infection, we profiled miRNA expressions in MDSCs isolated from HCV patients and HS. Among the miRNAs analyzed, 6 were significantly upregulated, 6 were downregulated, and 362 miRNAs remained unchanged in MDSCs from HCV patients compared to HS [17]. Among the miRNAs that were significantly downregulated, miR-124 was significantly inhibited (Figure 2L), and its level negatively correlated with STAT3 expression (Figure 2M), as determined by RT-qPCR. While miR-124 showed a negative correlation with RUNXOR, RUNX1, Arg1, and iNOS, there were no significant differences between their expression levels (data not shown). Since the STAT3 level positively correlated with the expression of RUNXOR as well as RUNX1 (Figure 2H,K), and, since our previous studies revealed a regulatory role for miR-124/STAT3 pathway in MDSC development during HCV infections [18], these new findings suggest a link between the RUNXOR/RUNX1 axis and the STAT3/miR-124 pathway that could play an important role in the regulation of MDSCs during chronic HCV infection.

### 3.5. RUNXOR and RUNX1 Regulate Each Other’s and Control miR-124 Expression via the STAT3 Signaling in MDSCs during HCV Infection

**Figure 3:**
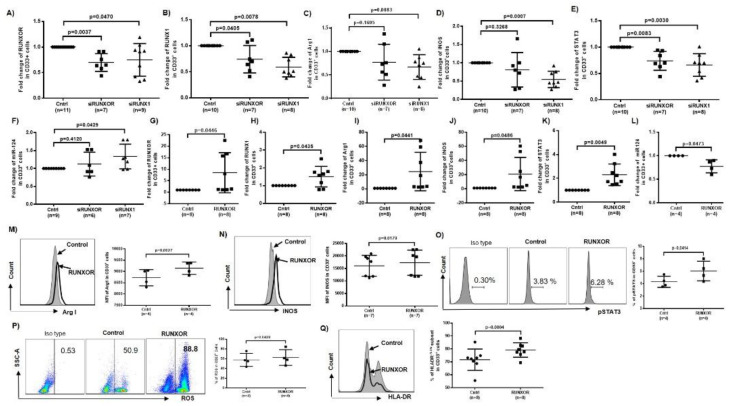
Silencing or Ectopic expression of RUNXOR or RUNX1 in CD33^+^ cells affects MDSC differentiation and immunosuppressive functions. (**A**,**B**) RUNXOR or RUNX1 expressions in HCV-CD33^+^ cells transfected by control, RUNXOR, or RUNX1 siRNA, determined by real-time RT-qPCR. (**C**–**F**) Arg1, iNOS, STAT3, and miR-124 expressions in HCV-CD33^+^ cells transfected with control, RUNXOR, or RUNX1 siRNA, determined by real-time RT-qPCR. (**G**–**L**) Ectopic expression of RUNXOR promotes RUNXOR, RUNX1, Arg1, iNOS, and STAT3 mRNA expressions and reduces miR-124 levels in CD33^+^ myeloid cells. (**M**–**Q**) Overexpression of RUNXOR in healthy CD33^+^ cells enhances Arg1, iNOS, and pSTAT3 protein expressions, increases ROS productions, and promotes immature myeloid cells (HLA-DR^−/low^ CD33^+^) differentiation into MDSCs, as determined by flow cytometry analysis.

To further elucidate the cause–effect relationships between RUNXOR/RUNX1 and miR-124/STAT3 expressions in MDSCs, HCV-derived CD33^+^ cells were transfected with RUNXOR or RUNX1 siRNA, respectively, followed by measuring their expression by RT-qPCR. As shown in Figure 3A, RUNXOR levels were significantly downregulated two days post-transfection with RUNXOR siRNA. Interestingly, silencing RUNX1 also downregulated the expression of RUNXOR in these cells. Similarly, silencing RUNXOR or RUNX1 downregulated the expression of RUNX1 (Figure 3B), indicating a positive feedback loop between these two regulatory molecules. Silencing RUNXOR expression attenuated the upregulation of Arg1, iNOS, and STAT3 and increased miR-124 levels. Only STAT3 level was significantly changed upon RUNXOR silencing, whereas the levels of these molecules were all significantly altered by silencing RUNX1 expression (Figure 3C–F).

**Figure 4:**
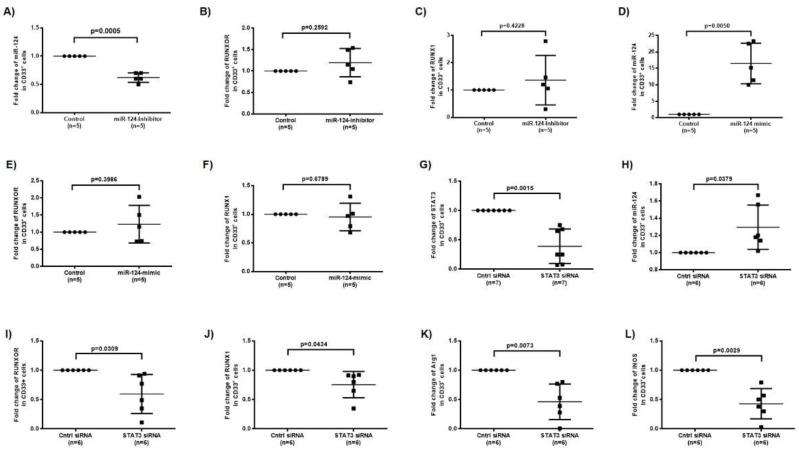
The RUNXOR and RUNX1 expressions correlate with the STAT3–miR124 axis in MDSCs during HCV infection. (**A**–**C**) Silencing miR-124 in HS-CD33^+^ cells significantly reduces the level of miR-124 but only slightly increases RUNXOR and RUNX1 expressions. (**D**–**F**) Overexpression of miR-124 in HCV-CD33^+^ cells significantly increases miR-124 levels but does not affect RUNXOR and RUNX1 expressions. (**G**–**L**) Silencing STAT3 expression in HCV-CD33^+^ cells significantly decreases STAT3 level, increases miR-124 expression, and inhibits RUNXOR, RUNX1, Arg1, and iNOS mRNA expressions (**I**–**L**).

To further demonstrate the relationships between RUNXOR/RUNX1 and miR-124/STAT3, CD33^+^ cells from HS were transfected with a RUNXOR CRISPR-Cas9 overexpression system, and the levels of RUNXOR, miR-124, and mRNAs of RUNX1, Arg1, iNOS, and STAT3 were measured as described above. As shown in Figure 3G–L, overexpression of RUNXOR significantly increased the mRNA expressions of RUNXOR, RUNX1, Arg1, iNOS, and STAT3, but reduced the level of miR-124. Flow cytometry analysis revealed that the mean fluorescence intensity (MFI) of Arg1, iNOS, and pSTAT3 protein levels, and production of ROS were also upregulated by the ectopic expression RUNXOR in healthy CD33^+^ cells (Figure 3M–P). Most importantly, overexpressing RUNXOR in healthy CD33^+^ cells also resulted in a significant increase in the frequency of HLA-DR^−/low^ immature (suppressive) cell subset within the CD33^+^ cell population (Figure 3Q). These results demonstrate that RUNXOR regulates the MDSC development and functions by modulating the expression of suppressive molecules. To investigate whether miR-124 regulates RUNXOR and RUNX1 expressions in MDSCs, we transfected healthy CD33^+^ cells with miR-124 inhibitor and measured the expressions of these molecules. As shown in Figure 4A–C, while miR-124 expression was significantly inhibited, RUNXOR and RUNX1 expressions were only slightly upregulated, and these alterations were not significantly different. In parallel, we increased miR-21 levels by transfecting CD33^+^ cells derived from HCV patients with miR-124 mimic and then measured the expressions of these molecules. As shown in Figure 4D–F, while the level of miR-124 was significantly increased, RUNXOR and RUNX1 levels were not significantly changed by miR-124 overexpression. These results suggest that miR-124 is likely a downstream target of the RUNXOR–RUNX1 pathway in MDSCs, because RUNXOR–RUNX1 could regulate miR-124 but not vice versa.

To determine whether RUNXOR regulates miR-124 via STAT3 signaling in MDSCs, we silenced STAT3 expression in CD33^+^ cells from HCV patients and measured the expressions of RUNXOR, RUNX1, and those suppressive molecules. Indeed, silencing STAT3 with siRNA significantly reduced STAT3 expression (Figure 4G) but upregulated the expression of miR-124 significantly (Figure 4H), which is in line with our previous report that STAT3 negatively regulates miR-124 expression in MDSCs [16]. Interestingly, silencing STAT3 expression also reduced the levels of RUNXOR, RUNX1, Arg1, and iNOS expressions (Figure 4I–L), indicating an important positive feedback loop involving STAT3 in the regulation of the RUNXOR–RUNX1–miR-124 axis and MDSC suppressive functions during HCV infection. Taken together, these results suggest that RUNXOR and RUNX1 positively regulate each other to control the expression of STAT3–miR-124 and immunosuppressive molecules in MDSCs. While STAT3, but not miR-124, can regulate the RUNXOR–RUNX1 axis in a positive feedback mechanism and negatively regulate miR-124 expression, this regulatory network acts in concert to promote MDSC development and suppressive functions during chronic HCV infection.

### 3.6. HCV-Associated Exosomes Regulate RUNXOR, RUNX1, and Suppressive Molecule Expressions in MDSCs

**Figure 5:**
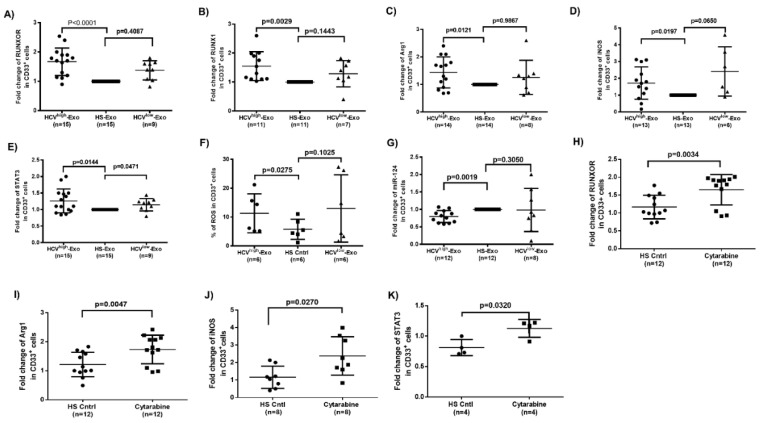
HCV-Exo or cytarabine treatment regulates lncRNA, miRNA, and mRNA expressions in MDSCs in vitro. (**A**–**G**) Expressions of RUNXOR, RUNX1, Arg1, iNOS, STAT3, ROS, and miR-124 in CD33^+^ cells treated with exosomes isolated from plasma of HCV-infected individuals (HCV^high^-Exo or HCV^low^-Exo) and HS (HS-Exo). (**H**–**K**) Cytarabine treatment induces the expression levels of RUNXOR, Arg1, iNOS, and STAT3 in HS-CD33^+^ cells.

To determine whether HCV-associated exosomes (HCV-Exo) can induce the alterations we observed in RUNXOR, RUNX1, and suppressive molecule expressions in MDSCs during HCV infection, we isolated exosomes from the plasma of HCV subjects with high or low viral load (HCV RNA = 17,000,000 or 17,000, named as HCV^high^-Exo and HCV^low^-Exo, respectively) and HS (HS-Exo). These exosomes were added to cultures of healthy PBMCs for five days, followed by the selection of CD33^+^ cells from the treated PBMCs. Similar to the observations in CD33^+^ cells isolated from HCV and HS, RT-qPCR analysis showed that, while both HCV^high^-Exo and HCV^low^-Exo induced RUNXOR (Figure 5A) and RUNX1 (Figure 5B) expressions, only HCV^high^-Exo led to a significant increase in both RUNXOR and RUNX1 expressions in treated cells. Moreover, HCV^high^-Exo significantly upregulated the expression levels of Arg1 (Figure 5C), iNOS (Figure 5D), STAT3 (Figure 5E), and ROS production (Figure 5F), while downregulated miR-124 levels (Figure 5G). These results suggest that HCV-Exo plays a role in the differential regulation of RUNXOR, RUNX1, STAT3, and miR-124 expressions during chronic HCV infection.

### 3.7. Cytarabine Regulates RUNXOR and Immunosuppressive Molecules in Healthy CD33^+^ Cells

Cytarabine, also known as cytosine arabinoside (Ara-C), is an antileukemic drug that treats AML and has been shown to increase the expression of RUNXOR [29]. We used cytarabine as a tool to manipulate RUNXOR expression in CD33^+^ cells to investigate its role in MDSC development. As shown in Figure 5H, treatment of healthy CD33^+^ cells with Cytarabine significantly increased RUNXOR expression. Notably, Cytarabine exposure also increased the expression of suppressive molecules Arg1, iNOS, and STAT3 (Figure 5I–K). These results further support the role of RUNXOR in promoting MDSCs and their suppressive functions.

### 3.8. Silencing RUNXOR and RUNX1 Expressions Reduce MDSC Frequencies and Suppressive Functions

**Figure 6:**
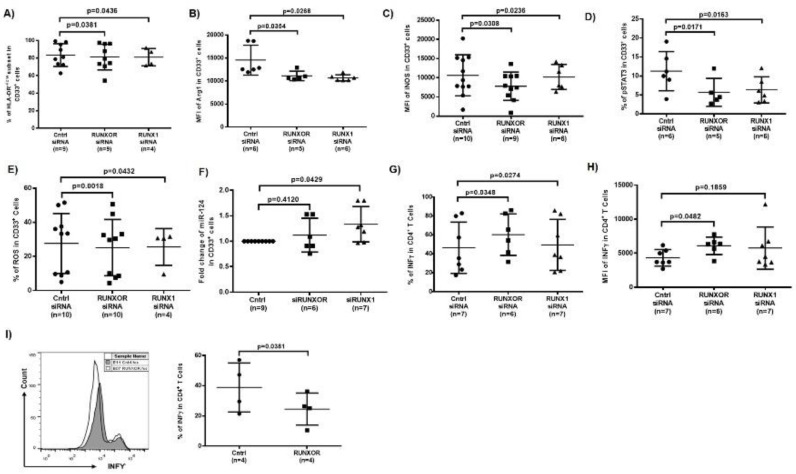
Silencing RUNXOR or RUNX1 inhibits the differentiation of MDSCs and their suppressive functions. (**A**) Silencing of RUNXOR or RUNX1 in HCV-CD33^+^ cells reduces the maturation of myeloid cells (HLA-DR^−/low^ subset in CD33^+^). (**B**–**F**) Silencing RUNXOR or RUNX1 in HCV-CD33^+^ decreases the Arg1, iNOS and pSTAT3 and increases miR-124 expression. (**G**,**H**) The percentage and MFI of IFN-γ production is restored in HCV-CD4 T cells following incubation with autologous CD33^+^ cells after silencing RUNXOR or RUNX1 expression. (**I**) IFN-γ production is suppressed in CD4 T cells following incubation with autologous HS-CD33^+^ cells overexpressing RUNXOR.

To determine whether silencing RUNXOR or RUNX1 expression attenuates HCV-induced MDSC expansion and immunosuppression, we transfected HCV-derived CD33^+^ cells with RUNXOR or RUNX1 siRNA, followed by measuring the frequencies of MDSCs and the expressions of immunosuppressive molecules in these cells. Compared to the control siRNA, RUNXOR and RUNX1 siRNA significantly reduced the frequencies of HLA-DR^−^, immunosuppressive cell subset within the CD33^+^ cells (Figure 6A), and concurrently decreased the levels of Arg1 (Figure 6B), iNOS (Figure 6C), pSTAT3 (Figure 6D), and ROS production (Figure 6E). Silencing RUNXOR and RUNX1 also increased the levels of miR-124 expression in CD33^+^ cells derived from HCV patients (Figure 6F). Importantly, the frequency and MFI of IFN-γ production by autologous CD4 T cells were significantly improved after culturing them with HCV-derived CD33^+^ cells that were transfected with RUNXOR and RUNX1 siRNA (Figure 6G,H). These results indicate that inhibiting the RUNXOR–RUNX1 pathway can attenuate MDSC differentiation and suppressive functions.

To further elucidate the role of RUNXOR-regulated MDSCs in T cell functions, we overexpressed RUNXOR in healthy CD33^+^ cells and then co-cultured them with autologous CD4 T cells for three days, followed by measuring IFN-γ production in activated CD4 T cells. As shown in Figure 6I, IFN-γ production was significantly suppressed. These results support a role for RUNXOR in promoting MDSC immunosuppressive effects on T cell functions.

## 4. Discussion

**Figure 7:**
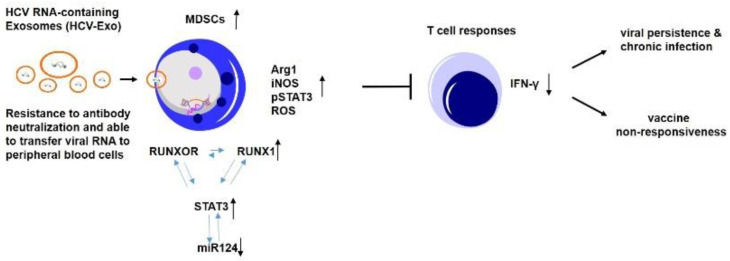
A model depicting the role of RUNXOR/RUNX1 in MDSC differentiation and suppressive functions. A model describing the role of RUNXOR–RUNX1 axis in MDSC development and the mechanism by which it suppresses host immune responses through regulating STAT3/miR-124. HCV infection can induce MDSC differentiation and production of immunosuppressive molecules, such as Arg1, iNOS, pSTAT3, and ROS, via induction of the RUNXOR–RUNX1 axis. RUNXOR and RUNX1 can reciprocally regulate each other’s expression and regulate STAT3–miR124 axis, which in turn promotes MDSC differentiation and its suppressive functions. This path will lead to the inhibition of T cell responses, thus potentially contributing to viral persistence and vaccine non-responsiveness during HCV infection.

MDSCs have been shown to expand and inhibit host immune responses in multiple disease models; however, the mechanisms of MDSC development during viral infection remain incompletely understood. In this study, we demonstrate that: (1) the expressions of lncRNA RUNXOR and its target gene RUNX1 are upregulated in MDSCs that accumulate in the peripheral blood of patients with chronic HCV infection; (2) the upregulation of RUNXOR–RUNX1 is positively associated with the levels of immunosuppressive molecule expressions in MDSCs, including Arg1, iNOS, STAT3, and ROS production, but negatively correlated with the miR-124 decline in these cells; (3) RUNXOR upregulation can be induced by HCV-Exo or Cytarabine exposure, which can also increase the frequency of MDSCs and the expression of immunosuppressive molecules, and decrease the level of miR-124 in MDSCs; and (4) silencing of RUNXOR or RUNX1 in MDSCs derived from HCV-infected subjects decreases MDSC immunosuppressive functions, whereas overexpressing RUNXOR in healthy myeloid cells has the opposite effect. Based on these findings and our previous studies [15,16,17,18], we propose a model (as depicted in Figure 7), illustrating the role of RUNXOR and the mechanism involved in promoting MDSC development to suppress the host immune response. According to this model, HCV-Exo can induce CD33^+^ cells differentiation into MDSCs and subsequently increase their production of immunosuppressive molecules, such as Arg1, iNOS, pSTAT3, and ROS via regulating the RUNXOR–RUNX1–STAT3–miR-124 axis. RUNXOR and RUNX1 can regulate each other’s expression in a positive feedback loop to regulate the STAT3–miR-124 signaling path, which in turn controls the MDSC development and suppresses T cell functions. While many components of the innate and adaptive immune responses play a role in viral infection and persistence, this novel mechanism of MDSC control is highly likely to be hijacked by immunomodulating viruses such as HCV in order to survive and induce viral persistence and vaccine non-responsiveness. Therefore, interrupting the RUNXOR–RUNX1–STAT3–miR-124 axis may serve as a potential immunologic approach to attenuate MDSC expansion and restore T cell functions in conjunction with antiviral treatment for chronic HCV infection.

LncRNAs are important regulators of chromatin structure, affecting the epigenetic state and expression level of target genes through interactions with histone modifiers, chromatin remodeling complexes, transcriptional regulators, or DNA methylation machinery [19,20,21]. LncRNAs can act as scaffolds by recruiting activators or suppressors at target gene promoters in the nucleus and can also epigenetically regulate gene transcription by regulating histone modifications and chromatin remodeling. LncRNAs also act as a sponge for miRNAs in the cytoplasm and help modify gene expression at the post-transcriptional level [35,36]. In our study, we found that RUNXOR and its target protein RUNX1 can regulate the STAT3–miR-124 axis, thereby regulating the development and suppressive functions of MDSCs during HCV infection. Notably, previous studies have shown that HCV promotes STAT3 signaling to maintain chronic infection [46]. HCV induces STAT3 directly by interacting with the HCV core protein [47], and indirectly through non-structural protein 5A (NS5A) via ROS [48]. In current study, we show that STAT3 is regulated through the RUNXOR–RUNX1 axis, as demonstrated by the RUNXOR–RUNX1 knockdown experiments (Figure 6D). In addition, our results show that silencing STAT3 expression also reduced the levels of RUNXOR, RUNX1, Arg1, and iNOS expressions (Figure 4I–L). Moreover, it has been reported that low miR-124 levels induced by HCV core protein via DNMT1 promote ICC cell migration and invasion by targeting SMYD3 [49], indicating the complexity of the host immune system. Here, our data suggest a regulatory loop involving the RUNXOR–RUNX1 pathway and STAT3–miR-124 signaling in MDSC induction during HCV infection. These new findings further support our previous studies, which link MDSC expansion to the induction of STAT3–miR-124 pathway [17].

Our results suggest that RUNX1 is a target for positive regulation by RUNXOR. The RUNX1 gene is a Runt-related transcription factor, also known as AML1 (acute myelogenous leukemia 1), that regulates the expression of several important hematopoietic regulator genes [50,51,52,53,54], including genes regulating B-cell maturation [55], granulocyte differentiation, and megakaryocyte maturation [56]. The RUNX1 gene is frequently mutated in various hematological malignancies [57]. Both homozygous and heterozygous mutations take place in the full-length of RUNX1 protein that results in either a single amino-acid substitution in the DNA-binding domain or in a C-terminal truncation mutation, leading to the removal of all or part of the transcriptional activation domain [58,59,60,61]. Expansion of common myeloid progenitors and granulocyte-macrophage progenitor (GMP) pools were observed under RUNX1 deficiency that was rescued by the inactivation of Hmga2 [62]. Similarly, an increase in the susceptibility to AML development in conjunction with MLL-ENL and N-Ras in the absence of RUNX1 supported the role of RUNX1 as a tumor-suppressor [63,64]. Recently, however, RUNX1 was shown to function in a pro-survival manner in leukemogenesis, indicating the importance of RUNX1 expression in AML1-ETO and MLL-AF9 cells [65]. Taken together, these data indicate that RUNX1 acts in both an oncogenic and a tumor suppressor mode in a context-dependent manner [43,44,66,67,68,69,70,71,72,73,74]. In the current study, the pattern of RUNXOR expression is rather similar to that of the RUNX1 gene, lending support to our notion that the RUNXOR–RUNX1 axis is important for MDSC development and function.

How RUNXOR and RUNX1 control each other’s expression in a mutually exclusive manner is unclear. Interactions among lncRNAs, miRNAs, and mRNAs have been described previously [75]. The multilayered complexity of RNA crosstalk and competition may arise due to lncRNA regulating the expression of both neighboring genes and/or distant genes [76]. Interestingly, the RUNX genomic regions overlap with RUNXOR, suggesting that the lncRNA RUNXOR participates in the regulation of RUNX expression [28,29,30]. This study clearly shows that RUNXOR and RUNX1 regulate each other’s expression to control STAT3–miR-124 expressions in MDSCs, which supports a recent report showing that the STAT3–miR214 axis plays an essential role in MDSC development during HCV infection [17]. RUNX1 inhibits the expression of suppressor of cytokine signaling 3 (SOCS3), an important negative feedback regulator of the STAT3 signaling pathway [77]. These findings suggest that RUNX1 regulates STAT3 phosphorylation, in part through the modulation of SOCS3 expression [77,78]. Based on our results, we conclude that the RUNXOR–RUNX1 axis plays an important role in controlling MDSC expansion and suppressive functions by regulating the STAT3–miR-124 axis during HCV infection. The putative cooperation of the two ncRNAs, RUNXOR and miR-124, in MDSC development and HCV pathogenesis warrants further investigation.

A growing list of lncRNAs have been identified as bona fide transcriptional regulators, and many studies are investigating these noncoding transcripts as potential biomarkers and therapeutic targets in human diseases. To our knowledge, this is the first report showing that the RUNXOR–RUNX1–STAT3–miR-124 axis promotes MDSC development and immunosuppressive functions during chronic HCV infection. Therefore, targeting this axis may provide a novel approach for immunomodulation in conjunction with antiviral therapy to protect against the immune regulatory effects of human viral infections.

## Figures and Tables

**Table 1:** Demographic information of the study participants.

Subjects	Numbers	Age (Mean)	Gender (M/F)	Viral Load and Other Characteristics
HCV	50	29–65 (46)	39/11	17,000–17,000,000 IU/mL, 36GT1, 8GT2, 6GT3
HS	54	22–64 (33)	41/13	All the tested negative for HCV, HBV and HIV
